# Fused Smart Sensor Network for Multi-Axis Forward Kinematics Estimation in Industrial Robots

**DOI:** 10.3390/s110404335

**Published:** 2011-04-13

**Authors:** Carlos Rodriguez-Donate, Roque Alfredo Osornio-Rios, Jesus Rooney Rivera-Guillen, Rene de Jesus Romero-Troncoso

**Affiliations:** HSPdigital-CA Mecatronica, Facultad de Ingenieria, Universidad Autonoma de Queretaro, Campus San Juan del Rio, Rio Moctezuma 249, 76807 San Juan del Rio, Qro., Mexico; E-Mails: cdonate@hspdigital.org (C.R.-D.); jrivera@hspdigital.org (J.R.R.-G.); troncoso@hspdigital.org (R.J.R.-T.)

**Keywords:** forward kinematics, sensor network, sensor fusion, FPGA, industrial robot

## Abstract

Flexible manipulator robots have a wide industrial application. Robot performance requires sensing its position and orientation adequately, known as forward kinematics. Commercially available, motion controllers use high-resolution optical encoders to sense the position of each joint which cannot detect some mechanical deformations that decrease the accuracy of the robot position and orientation. To overcome those problems, several sensor fusion methods have been proposed but at expenses of high-computational load, which avoids the online measurement of the joint’s angular position and the online forward kinematics estimation. The contribution of this work is to propose a fused smart sensor network to estimate the forward kinematics of an industrial robot. The developed smart processor uses Kalman filters to filter and to fuse the information of the sensor network. Two primary sensors are used: an optical encoder, and a 3-axis accelerometer. In order to obtain the position and orientation of each joint online a field-programmable gate array (FPGA) is used in the hardware implementation taking advantage of the parallel computation capabilities and reconfigurability of this device. With the aim of evaluating the smart sensor network performance, three real-operation-oriented paths are executed and monitored in a 6-degree of freedom robot.

## 1. Introduction

Flexible manipulator robots have wide industrial applications, with handling and manufacturing operations being some of the most common [1–3]. High-precision and high-accuracy in robot operations require the study of robot kinematics, dynamics and control [4]. The aim of forward kinematics is to compute the position and orientation of the robot end effector as a function of the angular position of each joint [1]. The online estimation of the forward kinematics can contribute to improve the controller performance by considering the joints’ motion collectively. Therefore, the precision and accuracy of such information is essential to the controller in order to increase its performance in real robotic operations.

Commercially available motion controllers use a single sensor for each joint to estimate the robot’s angular position; the most common sensor is the optical encoder [5–11], which provides a high-resolution feedback to the controller. However, it only gives information on the servomotor position and any deformations caused by joint flexibilities cannot be monitored [6,12], decreasing the robot’s accuracy. This problem is more evident in open-chain robots. Moreover, the provided information is relative, which means that it is impossible to estimate the initial position of the robot. Another sensor that is widely used in the estimation of the angular position of the robot joints is the gyroscope; it provides a measurement of angular rate of change, requiring the accumulated sum over time to estimate the angular position. Despite the fact that they can detect some nonlinearities that cannot be estimated with encoders, the quantized, noisy signal causes accumulated errors when angular position is required [13–15]. Furthermore, the estimated angular position is relative, which does not permit one to know the initial angular position of the robot joints. A good sensor that provides an absolute measurement is the accelerometer and it can be used to estimate the robot angular position [5,16–20]. Nevertheless, the signal obtained is noisy and contains much information that needs preprocessing before being used [21].

Two main issues need to be solved when the robot forward kinematics is required: the problems of using a single sensor to estimate the angular position of the joints and the online estimation of the forward kinematics. In this perspective, sensor fusion techniques improve the accuracy of the monitored variables, but at the expense of high-computational loads [22], which complicate the online estimation of the forward kinematics. Some works combine sensor fusion techniques and forward kinematics estimation. For example, in [7] accelerometer and encoder signals are fused using a disturbance observer to compensate some nonlinearities and a reset state estimator for position estimation; experimentation is performed on a linear motor positioning system, which requires no forward kinematics estimation. Another work that fuses encoder and accelerometer signals is presented in [16], where the forward kinematics of two links in a 6-DOF robot is calculated; different versions of an extended Kalman filter are used for sensor fusion. However, the efficacy of the proposed algorithm is demonstrated offline. Other works attempt to fuse more than two different sensors. In [12] the fusion of encoder, accelerometer and interferometer through a Kalman filter is presented to estimate the position of a parallel kinematic machine, but the analysis is limited to one-axis movement. In [6] camera, accelerometer and gyroscope sensors are combined through a kinematic Kalman filter for position estimation of a planar two-link robot to facilitate the forward kinematics estimation. In [23,24] a hardware-software architecture for sensor network fusion in industrial robots is proposed. Multiple PCs to process all the data collected from the sensors and to control the robot are used. However, they use the sensor fusion to estimate the robot contact force and the forward kinematics are estimated only from the encoder signals.

Reported works note the limitations of using a single sensor to estimate robots’ forward kinematics. Besides, forward kinematics is limited to a couple of joints due to the equations’ complexity. Therefore, the online forward kinematics estimation problem for multi-axis robots still requires additional efforts. Due to this, a dedicated processor capable of filtering and fusing the information of several sensors would be desirable. Also, multi-axis forward kinematics estimation in an online fashion would be advantageous.

The contribution of this work is performed of two stages: the improvement of the sensing method of conventional motion controllers through proposal of an encoder-accelerometer-based fused smart sensor network. Furthermore, we propose a smart processor capable of processing all the sensed encoder-accelerometer signals so as to obtain online forward kinematics estimation of each joint of a six-degree-of-freedom (6-DOF) industrial robot. The smart processor is designed using field-programmable gate arrays (FPGA) owing to their parallel computation capabilities and reconfigurability. It is designed through the combination of several methods and techniques to achieve online operation. The smart processor is able to filter and to fuse the information of the sensor network, which contains two primary sensors: an optical encoder, and a 3-axis accelerometer; and then to obtain the robot forward kinematics for each joint in an online fashion. The sensor network is composed of six encoders and four 3-axis accelerometers mounted on the robot. An experimental setup was carried out on a 6-DOF PUMA (Programmable Universal Manipulation Arm) robot, demonstrating the performance of the proposed fused smart sensor network through the monitoring of three real-operation-oriented 3D trajectories. Moreover, additional experiments were carried out whereby the forward kinematics obtained with the proposed methodology is compared against the obtained through the conventional method of using encoders. An improvement in the measurement accuracy is found when the proposed methodology is used.

## 2. Methodology

The use of accelerometers on 6-DOF PUMA robots requires placing them adequately in specific positions. The combination of accelerometers and encoders make up the sensor network that needs to be processed in order to estimate the angular position of each joint and the forward kinematics accurately. In this section, the placement of the sensor network on the PUMA robot is presented. Then, the FPGA-based forward kinematics smart processor is clearly described.

### 2.1. Sensor Network

**Figure 1.**
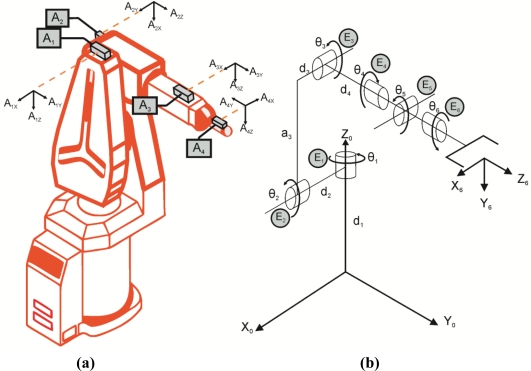
Sensor network location on the robot. **(a)** Accelerometer placement. **(b)** Schematic showing the parameters that describe the robot.

A sensor network is an array of diverse types of sensors to monitor several variables [25,26]; in this case the angular position of the joint flexibilities of the robot. The sensor network arranged on the robot is presented in Figure 1. Figure 1(a) depicts the position of the accelerometers on the robot. *A_ix_*, *A_iy_* and *A_iz_* are the measurements of each axis from the accelerometer *A_i_*. Figure 1(b) is a schematic of the robot including its link parameters. *θ_i_* represents the angular position of joint *i. a_i_* and *d_i_* represents the robot physical dimensions. Also, the localization of encoders *E_i_* is shown.

For this work, the forward kinematics estimation consists on the estimation of the joint angular position (*θ_i_*), the spatial position of each joint (*X_i_,Y_i_, Z_i_*); and the roll (*α_i_*), pitch (*β_i_*), and yaw (*γ_i_*) angles [1]. Such angles represent the rotation along the *Z*_0_,*Y*_0_,*X*_0_ axes, respectively, required to obtain the orientation of each joint.

#### 2.1.1. Angular Joint Position

The joint angular position is calculated with both encoder and accelerometer sensors. Afterwards, the obtained information is fused through a Kalman filter. In the case of the encoders, the angular joint position *θ_Ei_* can be calculated using Equation (1), where *E_i_* is the accumulated count given by encoder *i*; *SF* is a scale factor relating the minimum angular displacement required to obtain a count in the encoder, the units are rad/counts:

(1) $$\theta_{Ei}=\mathrm{SF}(E_{i})$$

Concerning the estimation of the angular joint position using accelerometers *θ_Ai_*, the corresponding equations are summarized in Table 1. In the case of the first angular position, the joint always moves perpendicularly to gravity force. Therefore, an accelerometer cannot detect the position changes in this joint. For this reason, only the encoder information is using for the angular position estimation in joint 1.

Such equations assume that the accelerometers provide a noise-free signal, which is unrealistic; thus, the signal requires a filtering stage before being used.

#### 2.1.2. Forward Kinematics

Forward kinematics provides the position and orientation (roll, pitch and yaw) of the robot through the angular position of each joint. In this case, the forward kinematics is calculated through the standard Denavit-Hartenberg notation [4]. The notation is a transformation matrix ^0^*T_i_* relating the reference coordinate frame (*Z*_0_,*Y*_0_,*X*_0_) with the coordinate frame of the joint *i* (*X_i_*,*Y_i_*,*Z_i_*). The notation requires obtaining the link parameters of the robot. Those parameters are the link length (*a_i_*), the link twist (*ϕ_i_*), the joint distance (*d_i_*) and the joint angle (*θ_i_*). Based on Figure 1(b), forward kinematics can be calculated through the link parameters presented in Table 2.

Those parameters are used to estimate the transformation matrix ^0^*T_i_*. The general form of the transformation matrix is presented in Equation (2):

(2) T0i=[MiDi01]

where *M_i_* [Equation (3)] contains the rotation information and *D_i_* is a vector containing the position of the link *i* [Equation (4)]:

(3) $$M_{i}=\left[\begin{array}{lll} m_{i,1,1} & m_{i,1,2} & m_{i,3,1}\\ m_{i,2,1} & m_{i,2,2} & m_{i,3,2}\\ m_{i,3,1} & m_{i,2,3} & m_{i,3,3} \end{array}\right]$$

(4) $$D_{i}=\left[\begin{array}{l} X_{i}\\ Y_{i}\\ Z_{i} \end{array}\right]$$

Therefore, forward kinematics can be calculated using Equation (3) and Equation (4). Position is directly estimated through Equation (4). Orientation can be estimated using Equations (5–7). Where *α_i_*,*β_i_*,*γ_i_* are the rotations along *Z*_0_,*Y*_0_,*X*_0_ axis, respectively:

(5) $$\;\alpha_{i}={\text{tan}}^{-1}\left(\sqrt{1-m_{i,3,1}^{2}-m_{i,1,1}^{2}}/m_{i,1,1}\right)$$

(6) $$\beta_{i}={\text{tan}}^{-1}\left(-m_{i,3,1}/\sqrt{1-m_{i,3,1}}\right)$$

(7) $$\gamma_{i}={\text{tan}}^{-1}\left(\sqrt{1-m_{i,3,1}^{2}-m_{i,3,3}^{2}}/m_{i,3,3}\right)$$

#### 2.1.3. Sequential Computation of Forward Kinematics

**Figure 2.**
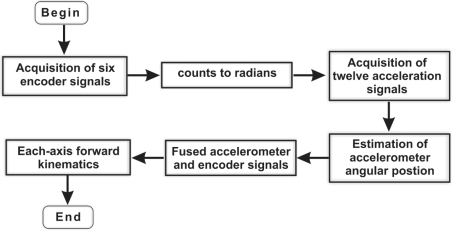
Flow diagram to estimate forward kinematics.

Encoders can provide a high-resolution measurement, but they are not capable of detecting some nonlinearities. Conversely, accelerometers, mounted on the robot, can detect some joint flexibilities and they provide an absolute measuring of orientation as well; however, the measurements are noisy and need filtering before being used. The fusion information of encoder and accelerometer takes the best of both sensors incrementing the accuracy of the monitored angular positions. A flow diagram that depicts the required sequential computing for sensor fusion and forward kinematics estimation is presented in Figure 2. The required operations are the acquisition of six encoder signals, conversion from encoder counts to radians, acquisition of twelve accelerometer signals, estimation of each-joint angular position, Fusion of encoder and accelerometer signals and the estimation of the forward kinematics for each axis.

Because of the amount of tasks that must be executed to obtain the forward kinematics estimation, conventional sequential processors are not suitable for the implementation since conventional industrial controllers require a measurement at a sampling period of 1 ms for conventional controllers, and 100 μs for high-speed controllers [27]. Moreover, evaluating the forward kinematics for each joint is not an easy task since the model complexity increases proportionally to the number of joints to be calculated [16].

### 2.2. FPGA-Based Forward Kinematics Smart Processor

Due to the amount of signals to be processed in order to obtain a robot’s forward kinematics a sequential processor is not recommendable for online operation. Conversely, FPGA provide significant advantages in signal processing due to their parallel computation capabilities and their reconfigurability [28] being helpful to process all the encoder-accelerometer signals online and to obtain the robot forward kinematics. Moreover, it is demonstrated that an algorithm implemented in FPGA processes 10–100 times faster than DSP and microprocessors [27]. For this reason the proposed smart processor is implemented in an FPGA.

**Figure 3.**
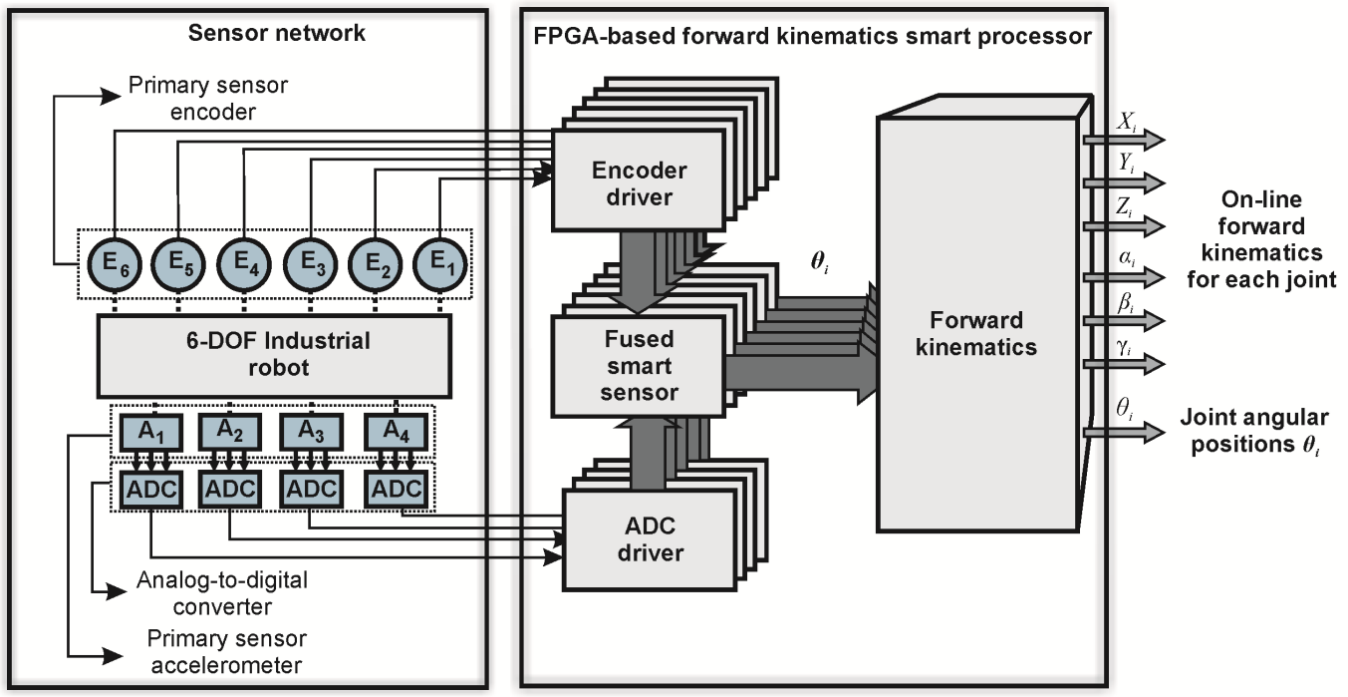
Sensor network and the FPGA-based forward kinematics smart processor.

A general block diagram depicting the interconnection of the sensor network with the FPGA-based forward kinematics smart processor is presented in Figure 3. Accelerometer information is digitalized by an analog-to-digital converter (ADC), and received by the smart processor through the ADC driver. Similarly, encoder information is managed by the smart processor through the encoder driver. Encoder and accelerometer information is filtered by arranged fused smart-sensor structures. Each structure filters and fuses the sensors information and estimates the joints angular position *θ_i_*. Subsequently, the information is used by the forward kinematics processor in order to obtain the forward kinematics of each joint online.

#### 2.2.1. Fused Smart Sensor

**Figure 4.**
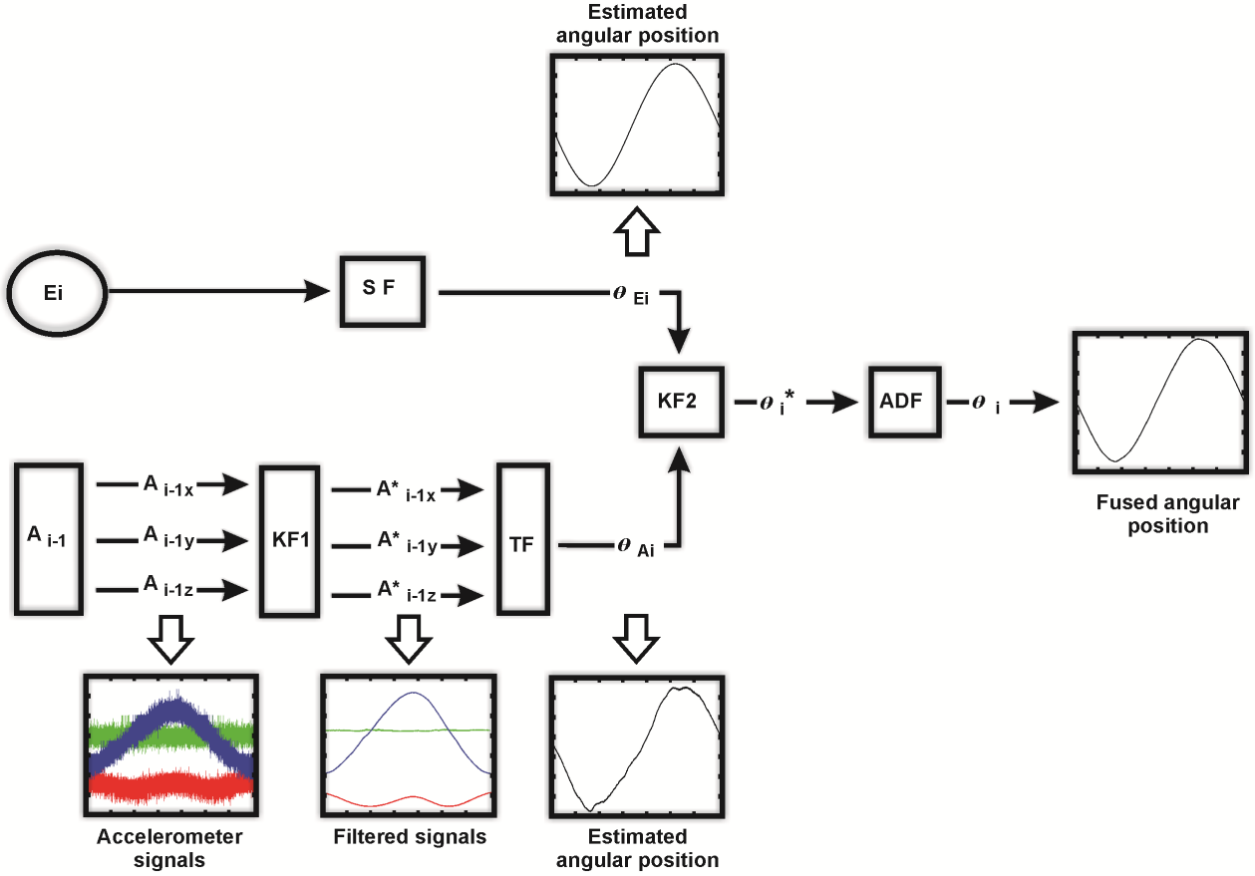
Basic fused smart sensor.

The fused smart sensor processes the information from encoder and accelerometer in order to obtain the angular position of each joint. An accelerometer, when mounted onto the joint of a flexible robot, contains information about the orientation with respect to gravity, plus vibrations and noise [5,29]. Only the orientation information from the accelerometer is required to estimate the angular position of each joint. Such information is located at low frequencies of the accelerometer bandwidth [21] requiring a filtering method and an additional processing unit to compute it. Filtering is a key factor to be considered since delays obtained with conventional filters are not permissible for online estimation. A method that has been proved to solve this issue is the Kalman filter [6,7,16,17,30]. Moreover, sensor fusion is also possible with this technique. Therefore, this algorithm is selected to filter and to combine encoder and accelerometer signals. The basic fused smart sensor structure is shown in Figure 4. Encoder *E_i_* and accelerometer *A*_*i*−1_ are oversampled, which helps to reduce the signal-to-quantization-noise ratio (SQNR) [21] and improves the filter response. The filtering stage (KF1) is performed by a Kalman filter, having the accelerometer signals as inputs (*A_i_*_−1_*_X_*,*A_i_*_−1_*_Y_*,*A_i_*_−1_*_Z_*). Next, filtered accelerometer signals ( $A_{i-1\;X}^{*},\;A_{i-1\;Y}^{*},\;A_{i-1\;Z}^{*}$) are sent to a processing unit TF where the angular position of the joint *θ_Ai_* is calculated. The encoder signal is processed in concordance with (1) in order to obtain the angular position *θ_Ai_*. After that, the joint angular position, estimated with encoder and accelerometer, are sent to a Kalman filter (KF2) where sensor fusion is executed. Finally, an average decimation filter (ADF) [21] is applied to the fused angular position $\theta_{i}^{*}$ to match the working sample frequency of the robot.

The equations for the Kalman filter are based on [31] and are described next. A Kalman filter works similarly to a feedback controller; the filter estimates the next state of the signal (predict) and then it obtains feedback in the form of noisy measurements to modify the predicted state (correct). General equations for the “predict” stage are presented in Equations (8) and (9):

(8) $$X_{k}^{*}=S\;X_{k-1}+Bu_{k-1}$$

(9) $$P_{k}^{*}=SP_{k-1}S^{T}+Q$$

Matrix *S* relates the previous state (*X_k_*_−1_) and the estimated actual state ( $X_{k}^{*}$), *B* relates an optional control input *u* with $X_{k}^{*}$, *Q* is the process covariance and $P_{k}^{*}$ is the *a priori* estimated error covariance.

In the case of the “correct” stage, the required equations are summarized in Equations (10–12), where *R* is the measurement noise covariance, *H* relates the measurements (*Z_k_*) with the current state *X_k_*, *K_k_* is a gain factor that minimizes the *a posteriori* estimated error covariance (*P_k_*):

(10) $$K_{k}=P_{k}^{*}H^{T}{\left(H\;P_{k}^{*}\;H^{T}+R\right)}^{-1}$$

(11) $$X_{k}=X_{k}^{*}+K_{k}\left(Z_{k}-H\;X_{k}^{*}\right)$$

(12) $$P_{k}=\left(I-K_{k}H\right)P_{k}^{*}$$

Concerning the filtering stage KF1, matrix *S* is an identity matrix; *B* = 0; $X={\left[A_{i-1X}^{*},A_{i-1Y}^{*},A_{i-1Z}^{*}\right]}^{T}$, *Z* = [*A_i_*_−1_*_X_*,*A_i_*_−1_*_Y_*,*A_i_*_−1_*_Z_*]*^T^*; *Q* is a diagonal matrix containing the covariance of each signal; likewise, *R* is a diagonal matrix with the noise covariance of each signal and *H* is an identity matrix. The processing unit TF differs for each joint of the robot. Required equations are summarized in Table 1.

Stage KF2 is a Kalman filter designed for the sensor fusion of two signals; in this case the parameters of the general Equations (8–12):*S* = 1; *B* = 0; $X=\theta_{i}^{*}$, *Z* = [*θ_Ei_*, *θ_Ai_*]*^T^*, *Q*, is the covariance of the angular position; *R* is a diagonal matrix with the noise covariance of each input signal and *H* = [1,1]*^T^*.

The averaging decimation filter is described in Equation (13), where *N* is a decimation factor relating the sampling rate of the sensors acquisition and the working sample frequency of the robot controller:

(13) $$\theta_{i}\left(k\right)=\frac{1}{N}\sum_{j=0}^{N-1}\theta_{i}^{*}\left(Nk-j\right)$$

#### 2.2.2. Forward Kinematics Hardware Structure

**Figure 5.**
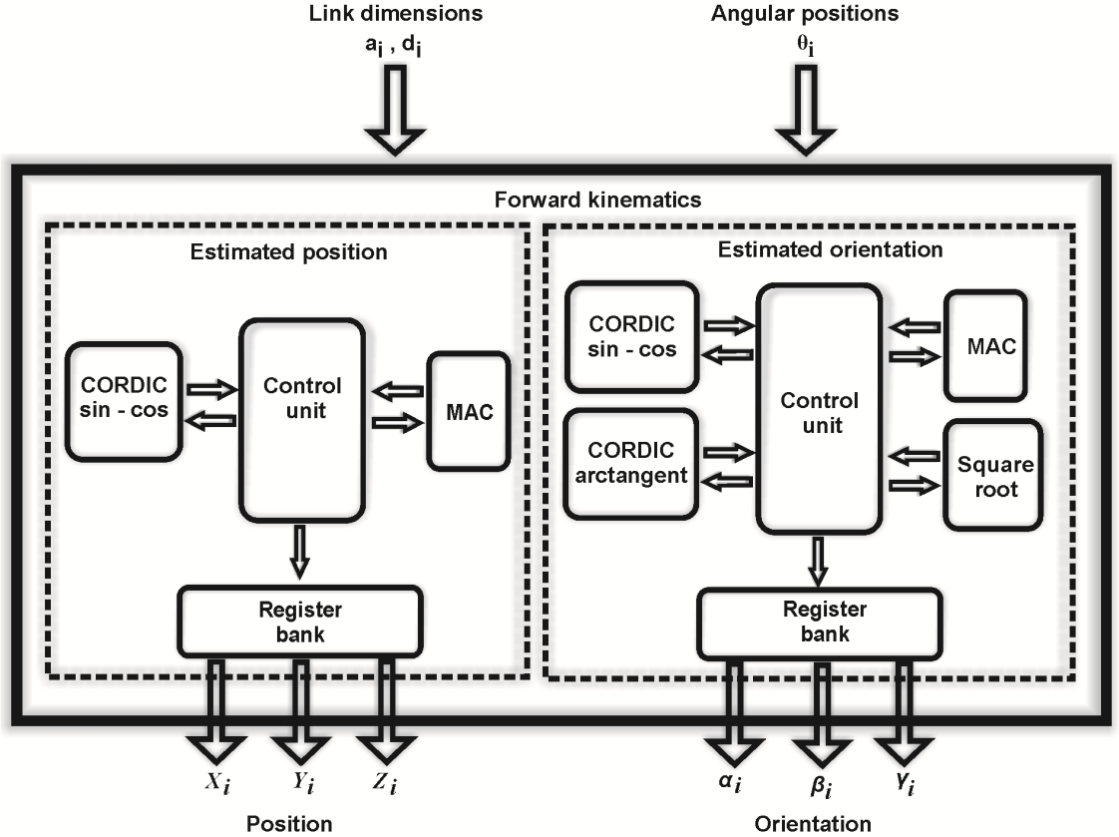
Forward kinematics hardware structure.

An important block of the smart processor is the block in charge of the forward kinematics (Figure 5). The input parameters are the link dimensions (*a_i_, d_i_*) and the angular position of each link (*θ_i_*), those parameters are used by two sub-processors to estimate position (*X_i_, Y_i_, Z_i_*) and orientation (*α_i_*,*β_i_*,*γ_i_*) of each link in concordance with Equations (4–7). The position estimator uses a multiplier-accumulator unit (MAC) and a coordinate rotation digital computer (CORDIC) configured to execute sine and cosine operations [32]; both are coordinated by a control unit that manages the operations performed for each unit. The estimated position is sent to a register bank. At the same time, the orientation estimator calculates the orientation of each joint. This processor has two embedded CORDIC units to estimate sine, cosine and arctangent functions. Also, a square root unit and a MAC unit are embedded. Alike the position estimator, a control unit coordinates the operations executed for each block and, when the orientation estimation is ready, the data is sent to the register bank.

## 3. Experiments and Results

In this section, the experimental setup and the results are presented for validating the proposed fused smart sensor network. The experimentation has three main objectives: firstly, a comparison of processing speed between a personal computer and the proposed smart processor with the aim of validating the use of FPGA-based parallel architectures against sequential processing. Secondly, the monitoring of three-real-operation oriented trajectories with the fused smart sensor network.

Finally, an experiment where the proposed methodology is compared with the methodology used by commercial controllers. In the first experiment, the proposed methodology is programmed in an FPGA using the digital structure presented in Figure 5. Then, it is also programmed in its sequential form and executed on a personal computer. The FPGA resource usage is shown and a time comparative between FPGA and personal computer is presented.

**Figure 6.**
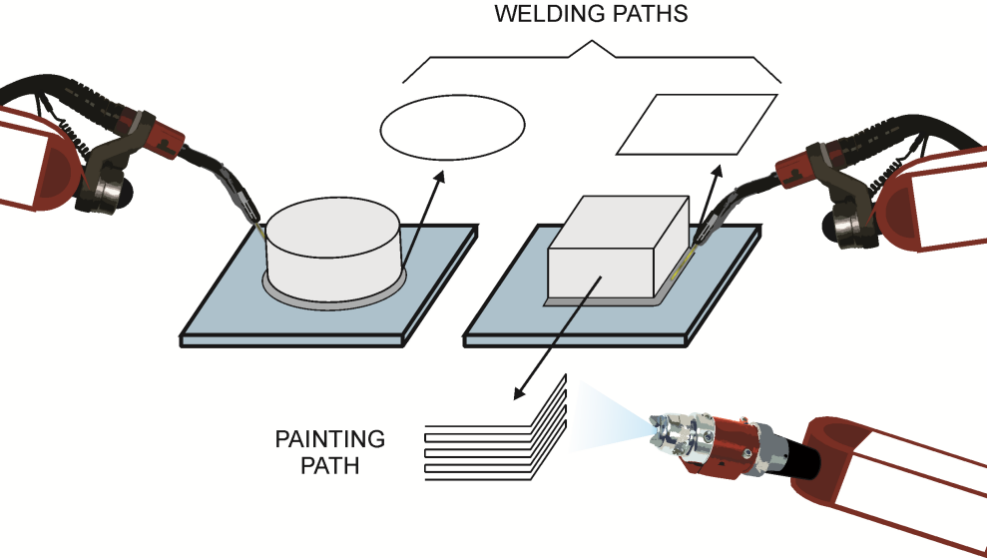
Real-operation-oriented paths.

In the second experiment, three paths are monitored through the smart sensor network. The online estimation of the angular position and the forward kinematics of each joint are performed on a 6-DOF PUMA robot. The monitored paths are selected considering the motion characteristics of real automatic robotic operations. Figure 6 shows the used paths and their relation with real operations in robots. Circle and square paths are selected considering welding joint operations, where lines and complex paths could be necessary. A zigzag path is designed considering a repetitive robotic operation such as painting. All the chosen paths have different complexity degree for being executed, due to the robot kinematics; the circle path has a low complexity degree, the square and zigzag path have higher complexity degree. Those paths are used to evaluate the robot controller performance.

Finally, in the last experiment the accuracy of the fused smart sensor network is evaluated. It consists of a comparison between two methods. First, the forward kinematics is calculated using high-resolution encoders (1,000 counts/rev). Next, the fused smart sensor network is used to calculate the forward kinematics. The errors of each sensing method are calculated through the methodology used in [33] where lasers are utilized to obtain an accurate measurement of the position and orientation of the robot.

### 3.1. Experimental Setup

**Figure 7.**
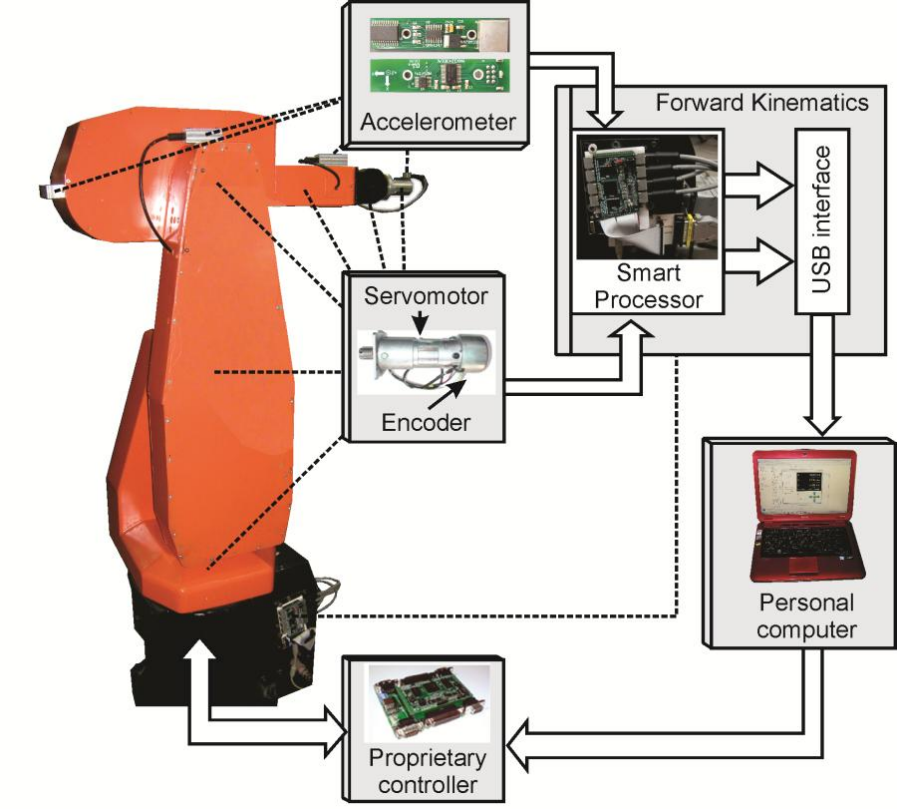
Experimental setup.

The experimental setup is shown in Figure 7, depicting the location of each accelerometer and the servomotors with the encoders. The used accelerometers are the LIS3L02AS4 units from STMicroelectronics [34] featuring measurements in three axis, a bandwidth of 750 Hz, a user-selectable full scale of ±2 g/±6 g (g = 9.81 m/s^2^), a 0.66 V/g sensitivity and a 5 × 10^−4^ resolution over a 100 Hz bandwidth. Accelerometer information is digitalized using 12-bit 4-channel ADS7841 ADC from Texas Instruments [35], with a maximum sampling rate of 200 kHz for the four channels. The signals obtained from the sensor network are sent to the smart processor to estimate the angular position and the forward kinematics for each joint. A USB interface unit is added to the smart processor in order to send the monitored forward kinematics to a personal computer to be visualized by the user. A proprietary controller [27] is used to control the robot at a sampling frequency of 1 kHz. The sampling frequency of the smart processor is set to 3 kHz.

### 3.2. Results and Discussion

In this section, the results of the proposed experimentation are presented. Also, the main advantages of the proposed methodology are discussed.

#### 3.2.1. Execution Time Comparative

The smart processor is implemented in a proprietary Spartan 3E XC3S1600E FPGA platform running at 48 MHz. Table 3 summarizes the resource usage of the FPGA after compilation.

The performance of the forward kinematics smart processor is compared with a personal computer (Sony Vaio VGN-CS170 featuring a two-core processor running at 2.26 GHz and 4 GB RAM). For each sample the smart processor requires 40 μs to calculate the complete forward kinematics, this processing time being suitable for conventional as well as high-speed servomotor controllers. On the other hand, the personal computer requires 21.22 ms to execute the same task. Therefore, the FPGA-based smart processor has the capability of online calculating the forward kinematics of a 6-DOF robot, while the PC is unable to perform the task online.

#### 3.2.2. Path Monitoring

**Figure 8.**
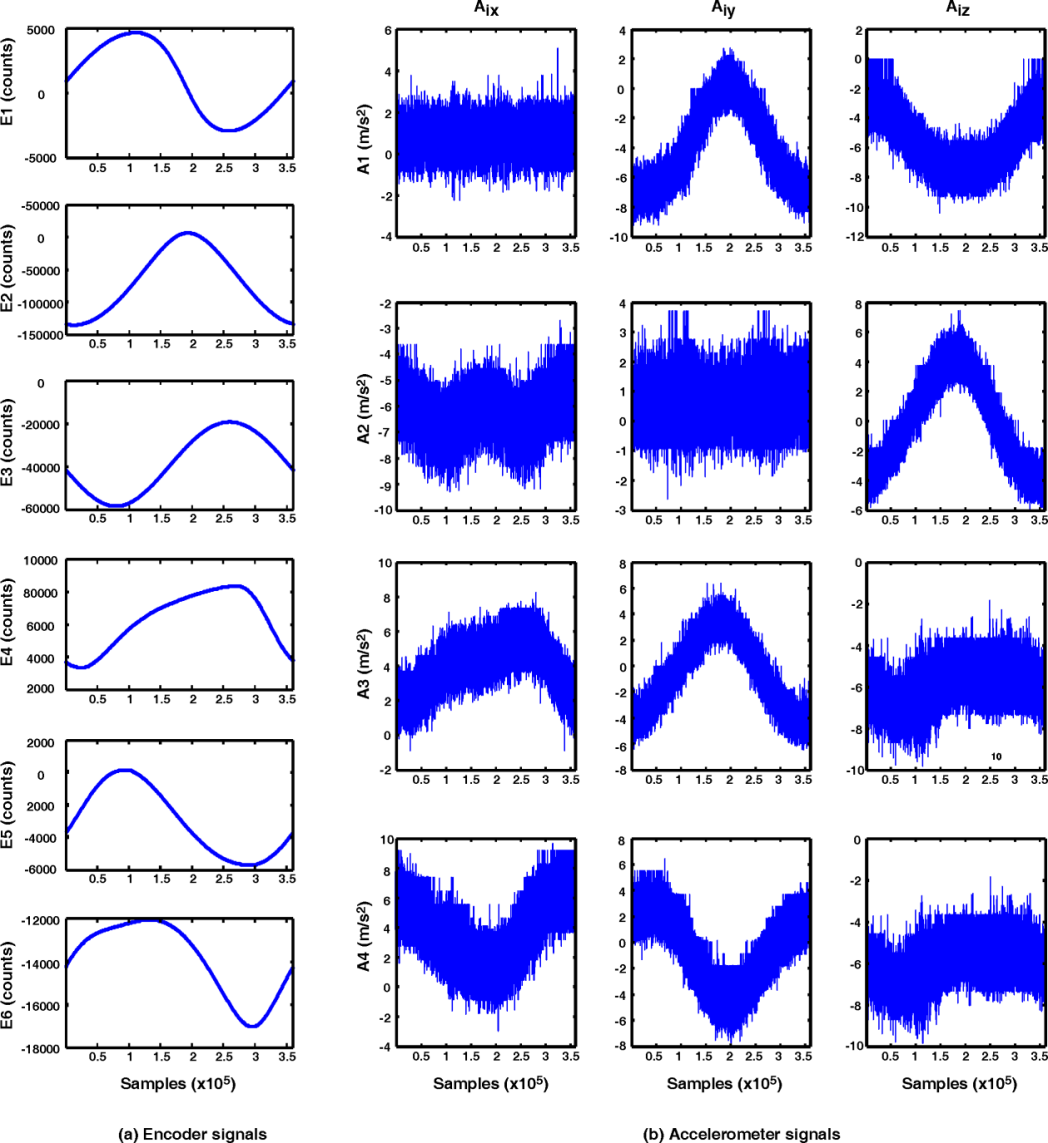
Monitored sensor network signals for the case of a circle path. **(a)** Encoder signals. **(b)** Accelerometer signals.

Encoder and accelerometer signals are monitored during the execution of the three paths in the 6-DOF PUMA robot. In Figure 8, the monitored encoder [Figure 8(a)] and accelerometer [Figure 8(b)] signals from the sensor network are presented for the case of the circle path. It can be observed that the encoder signals of each joint are noise free, due to their digital nature. Conversely, the accelerometers provide noisy measurements.

**Figure 9.**
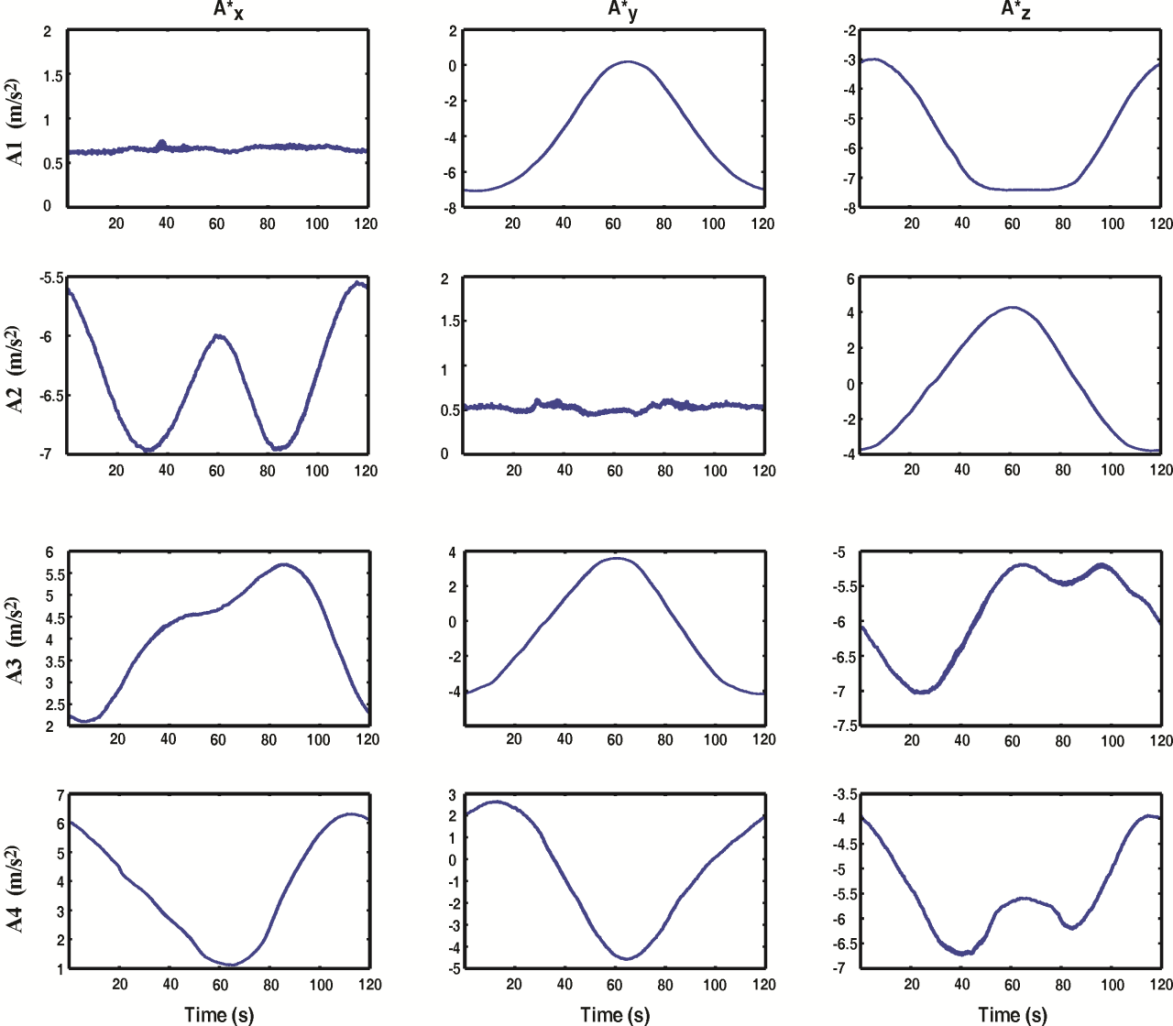
Filtered accelerometer signals.

The sensor network is composed of encoders and accelerometers that are processed by the FPGA-based forward kinematics smart processor, taking advantage of each sensor. Figure 9 shows the accelerometer signals after the filtering stage KF1, for the case of the circle path. The use of a Kalman filter for this purpose allows obtaining the filtered signals without delays.

**Figure 10.**
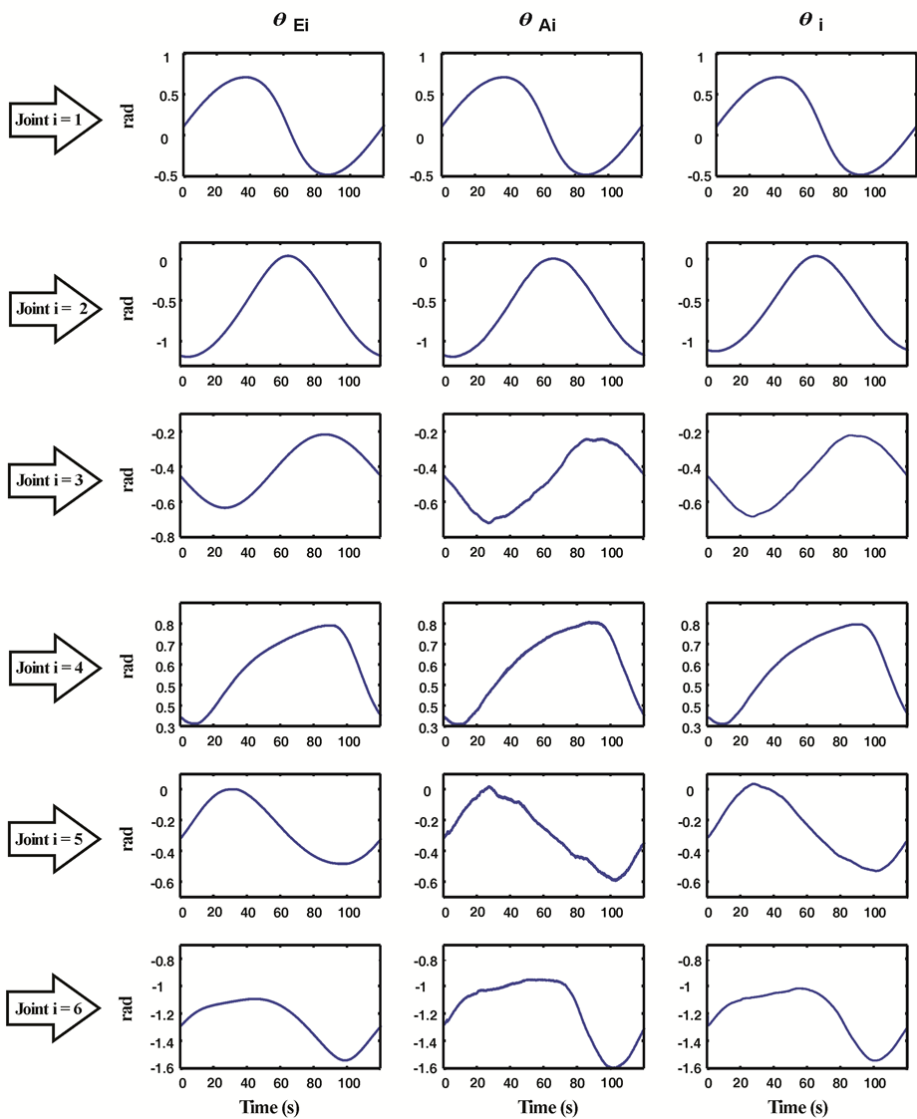
Angular position obtained with accelerometer and encoder, and fused estimation.

Figure 10 shows the angular position *θ_i_* obtained after the fusion and decimation of the angular position estimated with accelerometers *θ_Ai_* and the estimated with encoders *θ_Ei_*. Fused angular position takes the best of each sensor, the high resolution of encoders and the absolute measuring provided by accelerometers.

**Figure 11.**
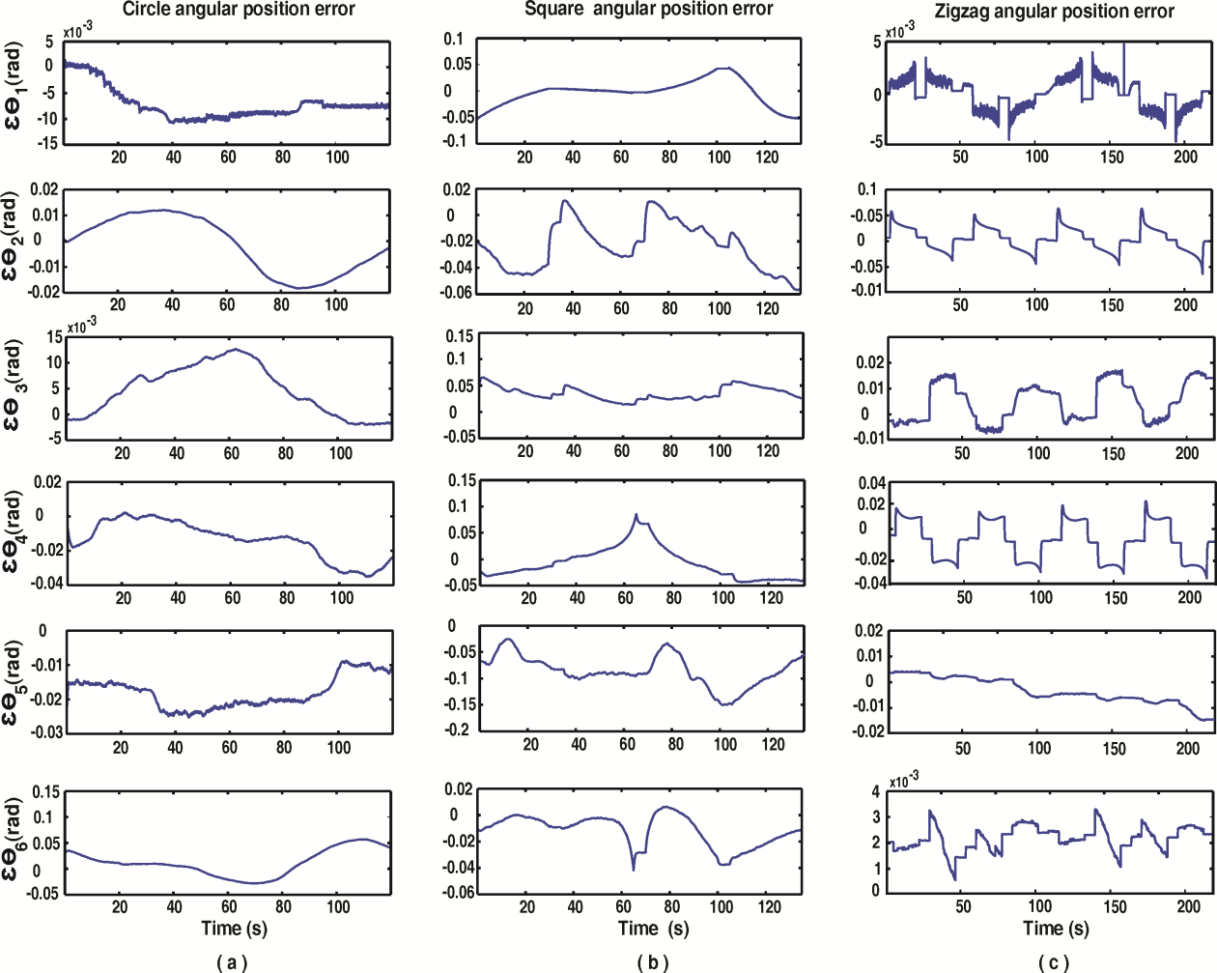
Monitored controller errors for each joint in the case of **(a)** circle path, **(b)** square path and **(c)** zigzag path.

With the aim of depicting the controller tracking monitored with the fused smart sensor network, Figure 11 shows the controller errors in each joint of the robot *ɛθ_i_*, when circle [Figure 11(a)], square [Figure 11(b)] and zigzag [Figure 11(c)] paths are executed.

As summarized in Table 4, the joint angular position errors *ɛθ_i_* are monitored with the fused smart sensor network. Both absolute and relative errors are shown for the case of circle, square, and zigzag paths. As it can be seen, the major problem is found in the square path movement, where joint 5 has the highest absolute and relative error. Such problem can occur because of the controller tuning or some mechanical problems in the robot.

**Figure 12.**
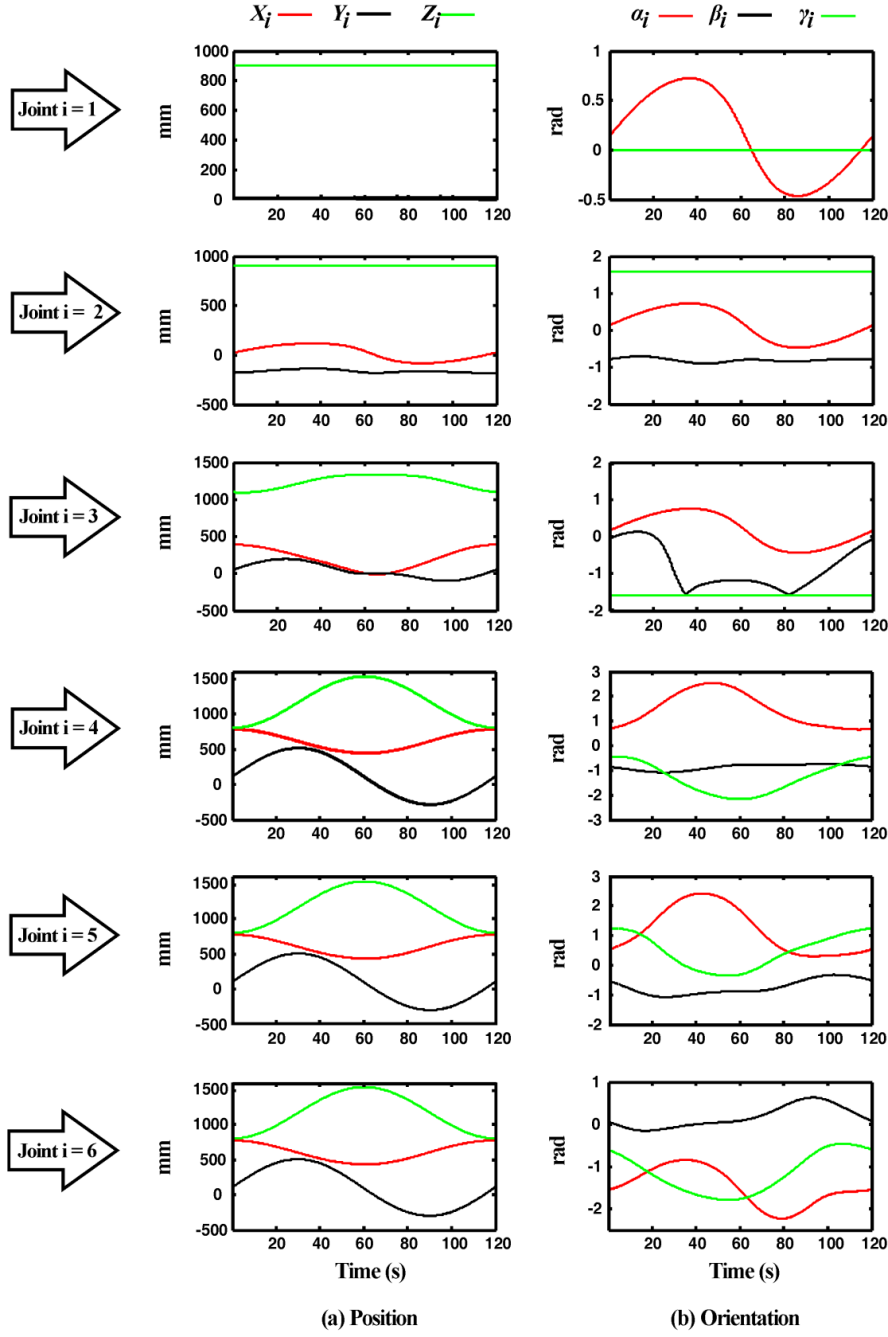
Each-joint forward kinematics **(a)** position, **(b)** orientation.

In Figure 12, the obtained angular position *θ_i_* and the dimensions (*a_i_*,*d_i_*), are used by the forward kinematics processor in order to obtain the position [Figure 12(a)] and orientation [Figure 12(b)] for each joint. The obtained data corresponds to the circle path.

**Figure 13.**
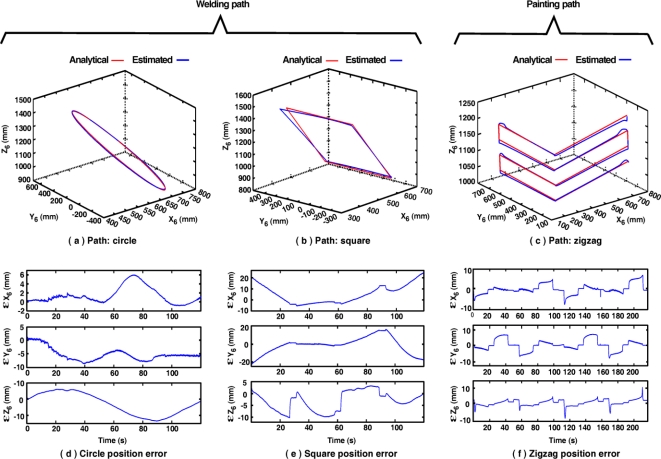
Analytical and estimated measures for welding and painting paths. **(a)** circle path, **(b)** square path, **(c)** zigzag path. Position error for **(d)** circle path, **(e)** square path and **(f)** zigzag path.

In order to evaluate the performance of the robot controller, the analytical end effector paths are compared with those measured with the fused smart sensor network. Figure 13 shows both the analytical and estimated path for the case of the circle [Figure 13(a)], square [Figure 13(b)] and zigzag paths [Figure 13(c)]. Errors between analytical and estimated paths for each axis (*ɛX*_6_,*ɛY*_6_,*ɛZ*_6_) are shown in Figure 13(d–e) for the case of the welding paths and Figure 13(f) in the case of painting path. Due to the characteristics of the fused smart processor network the proprietary controller errors are estimated and evaluated. Such errors fluctuate around 10 mm in the case of the circle path, 20 mm for the square path and 10 mm for the zigzag path.

**Figure 14.**
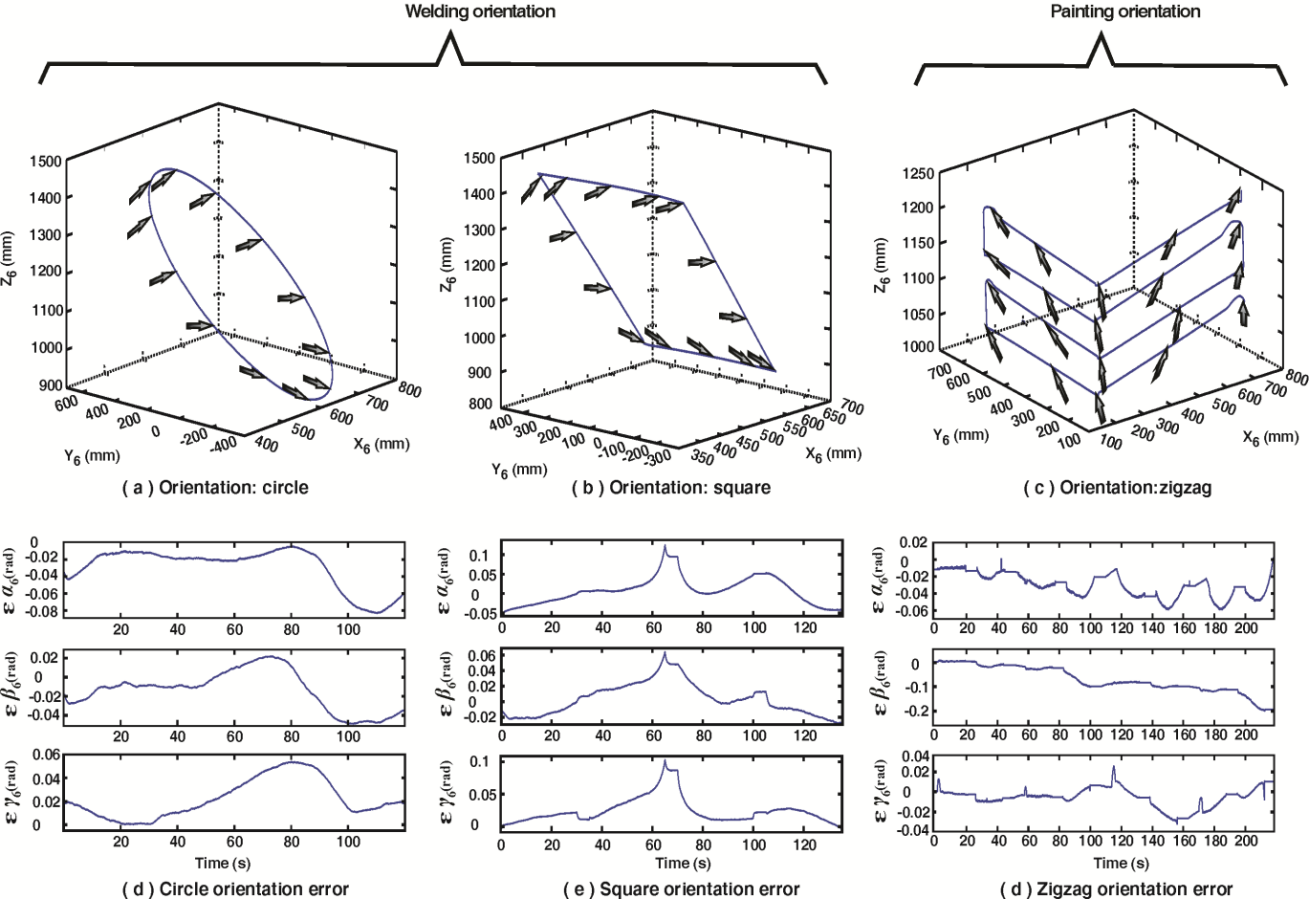
Monitored end effector orientation for welding and painting paths, **(a)** circle path, **(b)** square path, **(c)** zigzag path; Orientation errors for **(d)** circle path, **(e)** square path and **(f)** zigzag path.

As well as position, the orientation end effector accuracy is evaluated using the fused smart sensor network. A representation of the monitored orientation is shown in Figure 14(a) for the case of circle path, Figure 14(b) for the case of square path and Figure 14(c) for the zigzag path. Figure 14(d–e) show the orientation errors (*ɛα*_6_,*ɛβ*_6_,*ɛγ*_6_) for the circle, square and zigzag path respectively. Controller error oscillates around 0.08 rad for the circle path, around 0.1 rad in the case of the square path and 0.06 rad for the zigzag path.

The end effector position errors (*ɛX*_6_,*ɛY*_6_,*ɛZ*_6_) and orientation errors (*ɛα*_6_,*ɛβ*_6_,*ɛγ*_6_) are found with the fused smart sensor network are summarized in Table 5. The major problems are found in the square path, where the highest absolute and relative errors are measured. Such errors could be utilized by the controller and compensate them in order to increase the robot accuracy.

Three real-operation-oriented paths are analyzed in the experimentation showing the capability of the fused smart sensor network to perform an online estimation of the angular position and the forward kinematics of each joint of a 6-DOF PUMA Robot. A complete forward kinematics estimation would be advantageous to improve the robot performance, since the proposed fused smart sensor can evaluate the angular position of each joint *θ_i_* online, at the same time than position (*X_i_*,*Y_i_*,*Z_i_*) and orientation (*α_i_*,*β_i_*,*γ_i_*) are estimated.

#### 3.2.3. Methodology Comparative

The forward kinematics estimation obtained by using only encoders is compared with the forward kinematics estimation obtained by using the smart sensor network. To guarantee the repeatability of the measurements, the experiment is repeated 40 times. Table 6 shows the obtained relative errors. It is observed that the fusion of encoder and accelerometer provides a more accurate measurement when compared with the conventional encoder sensing.

Additionally, the proposed work is compared with other proposals through Table 7, where a comparative of the number of estimated parameters and the implementation features are shown. It can be observed that most of the work proposes a fusing method to improve the estimations. However, all the works limit their proposals to one or three DOF due to the complexity of evaluating forward kinematics in multi-axis robots [16] and the orientation information is not provided.

## 4. Conclusions

This work proposes a fused smart-sensor network for online estimation of the angular position and the forward kinematics of each joint in a 6-DOF PUMA robot. The smart processor collects the data sent from a sensor network composed by six encoders and four 3-axis accelerometers; this data is filtered and fused through Kalman filters. This guarantees that the joint angular positions are obtained without adding delays that are common in conventional filters. Moreover, owing to the reconfigurability and parallel computing of FPGA the proposed hardware structure features low execution time allowing the smart processor to calculate the robot forward kinematics of each joint online. The path monitoring shows the importance of fusing accelerometers and encoders signals to increment the accuracy of the forward kinematics estimation. The proposal allowed the evaluation of the robot controller performance through real-operation-oriented motions. Some errors that can be attributed to the controller tuning or some nonlinearities such as backlash and other structural deformations are found with the smart sensor network.

Furthermore, the methodologies comparative shows that the accuracy of the fused smart sensor is better when compared with the conventional encoder sensing. In addition, the comparison of features between the proposed smart sensor network and other reported works highlights the benefits of using the proposed methodology for the forward kinematics estimation in 6-DOF robots.

The proposed fused smart sensor network can be used in future research where the online estimated position of each joint can be used to feedback the controller in order to increase its performance. Moreover, additional sensors, such as gyroscopes and tilt sensors can be added to the sensor network in order to increase the precision of the monitored variables. Forward kinematics allows the estimation of the position and orientation of each joint, and most importantly, the end effector, in which the joints motion are considered collectively. This can be useful in industrial applications such as welding and painting operations, where precision and accuracy are mandatory.

## Figures and Tables

**Table 1.** Equations to estimate the angular position of each joint using accelerometers.

**Joint*i***	**Equation**
1	*θ_A_*_1_ = *θ_E_*_1_
2	*θ_A_*_2_*= π −* tan^−1^(*A*_1_*_y_*/*A*_1_*_z_*)
3	*θ_A_*_3_*=* tan^−1^(*A*_2_*_x_*/*A*_2_*_z_*)+ *θ_A_*_2_ − *π*
4	*θ_A_*_4_*= π −* tan^−1^(*A*_3_*_x_*/*A*_3_*_z_*)
5	*θ_A_*_5_*= π −* tan^−1^(*A*_4_*_y_*/*A*_4_*_z_*)− *θ_A_*_3_*+ θ_A_*_2_
6	*θ_A_*_6_*=* tan^−1^(*A*_4_*_x_*/*A*_4_*_z_*)− *π + θ_A_*_4_

**Table 2.** Link parameters of each joint.

*i*	*ϕ_i_*_−1_	*a_i_*_−1_	*d_i_*	*θ_i_*
1	0	0	*d*_1_	*θ*_1_*+ π*/2
2	*π*/2	*a*_3_	*d*_2_	*θ*_2_*+ π*/2
3	*π*	0	*d*_3_	*θ*_3_
4	*π*/2	0	*d*_4_	*θ*_4_
5	*π*/2	0	0	*θ*_5_
6	*π*/2	0	0	*θ*_6_

**Table 3.** FPGA resource usage.

**Element**	**Used**	**Available**	**Percentage (%)**
Slices	9,464	14,752	64
Slice Flip-Flops	6,617	29,504	29
4-input LUTs	13,357	29,504	45
Multipliers	12	36	33

**Table 4.** Measured angular position errors for each joint, for a circle and a square paths.

**Path**	**Measured error**	**Joint angular position errors**					
		*ɛθ*_1_	*ɛθ*_2_	*ɛθ*_3_	*ɛθ*_4_	*ɛθ*_5_	*ɛθ*_6_
Circle	Absolute (rad)	0.007	0.010	0.005	0.014	0.018	0.023
	Relative (%)	1.010	0.890	0.860	1.750	3.120	1.670
Square	Absolute (rad)	0.017	0.025	0.035	0.026	0.086	0.013
	Relative (%)	2.240	2.880	7.100	3.180	14.840	0.860
Zigzag	Absolute (rad)	0.001	0.012	0.177	0.070	0.051	0.021
	Relative (%)	0.100	1.290	1.950	7.430	1.640	0.190

**Table 5.** End effector measured errors in position and orientation for both circle and square path.

**End effector measured errors**		**Error type**	**Circle path**	**Square path**	**Zigzag path**
Position error	*ɛX*_6_	Absolute (mm)	1.562	16.034	1.817	
		Relative (%)	0.200	2.580	0.230
	*ɛY*_6_	Absolute (mm)	5.364	3.713	3.390
		Relative (%)	1.070	0.930	0.420
	*ɛZ*_6_	Absolute (mm)	6.303	29.379	2.568
		Relative (%)	0.410	2.020	0.210
Orientation error	*ɛα*_6_	Absolute (rad)	0.029	0.036	0.015	
		Relative (%)	1.300	1.560	4.600
	*ɛβ*_6_	Absolute (rad)	0.021	0.025	0.011
		Relative (%)	3.550	4.380	6.590
	*ɛγ*_6_	Absolute (rad)	0.020	0.137	0.006
		Relative (%)	1.110	7.680	0.960

**Table 6.** Comparative of errors between encoder and fused encoder-acelerometer.

**Sensing method**	**Error X-axis**	**Error Y-axis**	**Error Z-axis**	**Error***α*	**Error***β*	**Error***γ*
Fused Encoder and accelerometer	1.94%	3.81%	0.75%	29.36%	13.85%	53.01%
Encoder	2.59%	9.47%	4.00%	30.15%	55.70%	60.81%

**Table 7.** Features comparative between the proposal and reported works.

**Work**	**Sensing Method**	**Robot type**	**DOF**	**Position**	**Orientation**	**Online**
[6]	Vision sensor, gyroscopes and accelerometers	2-link planar robot	2	YES	NO	YES
[7]	Encoder, accelerometer and interferometer	Linear robot	1	YES	NO	YES
[12]	Accelerometer	Parallel kinematic machine	1	YES	NO	YES
[16]	Accelerometer, encoder	6-DOF robot	2	YES	NO	NO
[36]	Encoder	3-axis Cartesian manipulator	3	YES	NO	NO
This work	Accelerometer, encoder	6-DOF robot	6	YES	YES	YES
